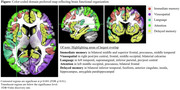# Assessment of Association of Tau PET Spatial Patterns with Cognitive Domains in Participants with Early Alzheimer's Disease in the Phase 2 Auτonomy Trial

**DOI:** 10.1002/alz70856_100821

**Published:** 2025-12-24

**Authors:** Ziad S. Saad, Ritobrato Datta, Maggie Fedgchin, Jennifer Bogert, Janice Wong, David Henley

**Affiliations:** ^1^ Johnson & Johnson, San Diego, CA, USA; ^2^ Johnson & Johnson, Titusville, NJ, USA; ^3^ Johnson & Johnson, Raritan, NJ, USA; ^4^ Johnson & Johnson, Cambridge, MA, USA

## Abstract

**Background:**

Posdinemab is an anti‐phosphorylated tau monoclonal antibody currently under investigation in early Alzheimer's disease (AD). The phase‐2 Auτonomy trial (NCT04619420) enrolled participants with mild cognitive impairment (MCI) or mild AD dementia (Clinical Dementia Rating‐Global Score [CDR GS] 0.5/memory box ≥0.5) with intermediate levels of tau, pre‐screened using a Janssen p217+tau plasma assay and confirmed with tau Positron Emission Tomography (PET). Repeatable Battery for the Assessment of Neuropsychological Status (RBANS) indices representing five cognitive domains were analyzed for voxelwise associations with tau PET. A brain wide map was reconstructed that reveals associations between cognitive domains and tau pathology.

**Method:**

Neurofibrillary tangle levels from 422 randomized participants were quantified using ^18^F‐MK‐6240 Standardized Uptake Value Ratio (SUVR; cerebellar gray reference region). SUVR volumes were non‐linearly aligned to Montreal Neurological Institute space and smoothed within a gray matter mask to a Full Width at Half Max of 12 mm. Associations with each cognitive domain were computed using Spearman rank to minimize outlier effect. For each of the 5 correlation volumes, voxel values were transformed to their rank. Finally, a domain preference map was generated by assigning to each voxel the highest ranked of the 5 domains and its corresponding Spearman correlation to enable statistical thresholding.

**Result:**

Correlations were significant (uncorrected *p* <10^‐3^, False Discovery Rate q<0.01) across most of the brain for all, but the visuospatial domain, for which significant correlations were largely in right occipital cortex. The following domain associations were observed (Figure): Visuospatial to right occipital cortex, with trends of association in left occipital cortex; Language to left temporal, supramarginal, and inferior parietal; Delayed‐memory to inferior temporal, fusiform, anterior cingulate, insula, and hippocampus; Attention to left middle occipital, precuneus, and bilateral frontal; Immediate‐memory to bilateral middle and superior frontal, precuneus and middle temporal.

**Conclusion:**

In participants with intermediate tau and early AD, randomized in the Auτonomy trial, we map using a spatially unbiased analysis of tau PET, which cognitive domains are relatively most impacted by tau pathology across the brain. The ensuing maps reflect established brain functional organization. These findings demonstrate close association between presence/location of tangles and cognitive profile.